# ROLE OF RELIGIOUSNESS AND SPIRITUALITY AND SOCIAL NETWORKS IN DETERMINING DEPRESSION AMONG OLDER KOREAN AMERICANS

**DOI:** 10.1093/geroni/igz038.1910

**Published:** 2019-11-08

**Authors:** Soonhee Roh, Yeon-Shim Lee, So-Young Park

**Affiliations:** 1 University of South Dakota, Sioux Falls, South Dakota, United States; 2 San Francisco State University, San Francisco, California, United States; 3 New York University, New York, New York, United States

## Abstract

Korean Americans (KAs) are one of the fastest growing minority populations in the U.S. Depression is the most common psychological problem among older KAs. While the relationship between religiousness/spirituality (R/S) and well-being in later life is an important health concern, older KAs are often affiliated with a protestant church and have the highest church participation. This study assessed the role of R/S and social networks in determining depressive symptoms and identified the best predictors of depressive symptoms. Data were drawn from a cross-sectional survey with 200 older KAs residing in New York. Best-subsets regressions were used to evaluate the best predictors of depression. Findings indicated that nearly 30% of older KAs reported experiencing mild or severe depressive symptoms. The best model fit for depression involved physical health, R/S coping skills, social networks, and annual household income. Social networks and R/S coping skills were found to be a protective factor against depressive symptoms and may be an effective tool for health care strategies in the management of depression and health-promoting behaviors. Careful assessment of R/S and social networks among older KAs may provide a more comprehensive approach to traditional, biomedically derived clinical practices by focusing on the whole person in early identification of risk factors, prognosis, and intervention for depression. Health education and intervention could be framed in ways that strengthen such psychosocial coping resources for older KAs. Facilitating social participation and mobilizing R/S resources in a wide range of personally meaningful activities may mitigate psychological distress and enhance life satisfaction.

